# Machine learning-based hybrid risk estimation system (ERES) in cardiac surgery: Supplementary insights from the ASA score analysis

**DOI:** 10.1371/journal.pdig.0000889

**Published:** 2025-06-23

**Authors:** Ayşe Banu Birlik, Hakan Tozan, Kevser Banu Köse

**Affiliations:** 1 Department of Healthcare System Engineering, Graduate School of Engineering and Natural Sciences, Istanbul Medipol University, Istanbul, Turkey; 2 Department of Medical Services and Techniques, Istinye University, Istanbul, Turkey; 3 College of Engineering and Technology, American University of the Middle East, Kuwait; 4 Department of Biomedical Engineering, School of Engineering and Natural Sciences, Istanbul Medipol University, Istanbul, Turkey; National Yang Ming Chiao Tung University, TAIWAN

## Abstract

Accurate prediction of postoperative mortality risk after cardiac surgery is essential to improve patient outcomes. Traditional models, such as EuroSCORE I, often struggle to capture the complex interactions among clinical variables, leading to suboptimal performance in specific populations. In this study, we developed and validated the Ensemble-Based Risk Estimation System (ERES), a machine learning model designed to enhance mortality prediction in patients undergoing coronary artery bypass grafting and/or valve surgery. A retrospective analysis of 543 patients was performed using six machine learning algorithms applied to preoperative clinical data to assess predictive accuracy and clinical outcomes. Feature selection techniques, including Gini importance, Recursive Feature Elimination, and Adaptive Synthetic Sampling, were employed to improve accuracy and address class imbalance. ERES, which utilizes 15 key features, demonstrated superior predictive performance compared to EuroSCORE I. Calibration plots indicated more accurate probability estimates, whereas SHAP analysis identified creatinine, age, and left ventricular ejection fraction as the most significant predictors. The decision curve analysis further confirmed the superior clinical utility of ERES across a range of decision thresholds. Additionally, although the American Society of Anesthesiologists (ASA PS) score had limited predictive power independently, its combination with EuroSCORE I enhanced the predictive performance. Integrating machine learning models like ERES into clinical practice can improve decision making and patient outcomes although external validation is warranted for broader implementation.

## Author summary

Accurately predicting mortality risk in patients undergoing cardiac surgery is critical for improving clinical outcomes and guiding preoperative decision-making. Traditional scoring systems such as EuroSCORE I, while widely used, often fall short in capturing the complex, non-linear relationships among patient variables. In this study, we developed the Ensemble-Based Risk Estimation System (ERES), a machine learning-based model that leverages preoperative clinical and laboratory data to more precisely estimate short-term mortality risk after coronary artery bypass and/or valve surgery. Using advanced techniques such as feature selection, data balancing, and model calibration, ERES outperformed both traditional models and other machine learning algorithms across multiple performance metrics. The model identified creatinine, age, and ejection fraction as key predictors of postoperative outcomes. Additionally, although the ASA score alone showed limited predictive ability, its integration with EuroSCORE I improved the overall model performance. Our findings suggest that machine learning models like ERES offer a more personalized and accurate approach to risk stratification, especially in complex or high-risk cases. We strongly believe that supporting studies like this will contribute to the development of more effective health policies and evidence-based clinical practices.

## Introduction

In cardiac surgery, especially for high-risk procedures such as Coronary Artery Bypass Grafting (CABG), accurately predicting patients’ surgical risks is critical importance [1]. The EuroSCORE system, which is widely used in Europe, is a significant scoring model developed to forecast early mortality risks among cardiac surgery patients [2]. Roques et al. identified mortality risk factors in adult patients undergoing cardiac surgery during the development of the EuroSCORE [3]. Currently, there are three versions of EuroSCORE: Standard EuroSCORE, Logistic EuroSCORE, and EuroSCORE II [3–5]. Hospitals in Turkey also implement a system based on this scoring model, known as Cardiac Risk Scoring, and determine hospital charges accordingly. Traditional risk models often rely on a certain (limited or excessive) number of predetermined parameters, which may cause critical information and their interactions to be overlooked, which may fully reflect the patient’s health status. Thus, the accuracy of the risk assessment may remain low. Such models often fail to adequately evaluate nonlinear interactions and exhibit limited performance in different clinical settings. [6]. In particular, in high-risk patients, these models may encounter discrimination and calibration issues, resulting in difficulty in making accurate predictions in individuals with complex pathologies. Despite these limitations, traditional models continue to provide critical criteria for assessing and improving hospital performance [7]. Advancements in big data analytics and machine learning (ML) algorithms can significantly enhance the capabilities of modern risk assessment tools. In the surgical context, there is an increasing need for personalized prediction systems tailored to specific patient groups, underscoring the importance of ML models. Linear models are limited in terms of predictive success because they are too dependent on the dataset on which they are trained, cannot model complex interactions between different variables, and cannot represent rare situations. As demonstrated in various applications of multi-criterion decision analysis and fuzzy logic-based models, structured decision-making frameworks are crucial in complex clinical environments [8,9]. The accuracy of the proposed model may decrease in datasets. In recent years, various ML models have been developed to predict the risk of clinical events. These approaches, by modeling nonlinear effects and analyzing more complex interactions, have the potential to overcome the limitations of linear models, offering better management of procedural heterogeneity and incomplete data [6,10].

In addition to EuroSCORE, another commonly used scoring system for surgical risk prediction is the American Society of Anesthesiologists (ASA) physical status classification. Established in 1963, the ASA score is a simple subjective measure of a patient’s overall health and comorbidities before surgery. Originally designed to estimate the overall mortality risk in surgical patients, the ASA score has been shown to more specifically predict cardiac and pulmonary complications [11]. Although it has been widely adopted and applied across various surgical disciplines, including cardiac surgery, the ASA score is inherently subjective, raising concerns about its reliability in predicting postoperative outcomes, especially in high-risk procedures [12]. Studies have shown that the ASA classification is significantly associated with postoperative morbidity and mortality, but its subjectivity limits its consistency as a reliable predictor when compared with more objective preoperative measures [13]. However, to address its limitations, the ASA score should be used in combination with other, more objective preoperative metrics for a more accurate assessment of postoperative outcomes. This approach allows for integrating broader clinical insights—derived from both subjective and objective data—leading to more comprehensive risk stratification. Combining the ASA score with models such as EuroSCORE, which incorporates specific clinical variables and surgical factors, can enhance predictive accuracy by compensating for the individual weaknesses of each system. Therefore, the complementary use of the ASA score alongside more quantitative models could provide a more robust framework for evaluating surgical risk, particularly in complex and high-risk patient populations.

In this study, we explored the potential to develop a regionally adapted hybrid ML-based model to more accurately predict short-term mortality risk using preoperative clinical and laboratory data in patients undergoing CABG and/or combined mitral or aortic valve surgery. Additionally, the predictive performance of the ASA classification and EuroSCORE systems in estimating postoperative mortality was compared among 179 cardiac surgery patients, and the combined model was tested to determine whether it provided superior predictive power compared with each individual score. This study builds upon and refines our previous research by improving the predictive models and enhancing their methodological robustness [14].

## Methods

### Study design and population

This retrospective study was conducted at Istanbul Medipol Mega University Hospital, Istanbul, Turkey, including adult patients (+18) who underwent CABG and/or combined mitral valve replacement (MVR) or aortic valve replacement (AVR) surgery. Since planning was assessment-oriented, accessible data were identified, and therefore only preoperative variables were included in the predictive model, excluding features that manifest during surgery. The study focused on patient data encompassing mortality rates within 30 days post-surgery, either during hospitalization or following discharge, comprising 543 patients. This observational study was conducted in accordance with the Declaration of Helsinki, as renewed in 2013. Due to the observational nature of the study, an informed consent was not obtained. Ethical approval was obtained from the Non-Interventional Clinical Research Ethics Committee (Decision No: 206; Reference: E-10840098-772.02-5254).

### Improvements in risk prediction: insights from our previous research

In our current study, building on findings from our previous research, we aimed to comprehensively examine the factors influencing CABG outcomes and implement significant improvements to enhance predictive performance. This study expanded the dataset, addressed missing data using appropriate imputation methods, and applied feature selection to identify key variables critical for model accuracy [14]. First, the dataset was expanded to provide a more robust foundation for the model. Missing values for categorical variables were imputed using the mode, whereas missing values for numerical variables were filled using the median. Additionally, the proportion of missing data for each variable was calculated, and variables with more than 20% missing values were excluded from the analysis to improve the model’s accuracy. To optimize the model’s performance, feature selection (FS) was applied to identify the most important variables. The selected features were then incorporated into the development of the hybrid algorithm. Among the key variables, creatinine (CR) and left ventricular ejection fraction (LVEF) were particularly prominent. The EuroSCORE was also treated as a numerical variable, with appropriate thresholds established to categorize risk levels.

Calibration curves were constructed to evaluate the clinical utility of the model, allowing for a more detailed assessment of its predictive accuracy. A net benefit analysis was also considered, particularly during this phase. Unlike our previous work, which only involved a dataset with categorical variables, this study utilized a mix of both numerical and categorical variables. In addition, to address class imbalance within the dataset, the Adaptive Synthetic Sampling (ADASYN) technique was employed, which resulted in a more balanced training dataset. These improvements aim to significantly enhance the model’s predictive performance and yield more reliable outcomes in clinical applications. Additionally, to more accurately predict postoperative mortality risk, a comprehensive analysis of the American Society of Anesthesiologists (ASA) score was conducted alongside EuroSCORE. Preoperative risk assessment is particularly critical in elderly patients with cardiovascular comorbidities. Although the ASA score is a widely used tool for quickly and effectively assessing a patient’s general health status before surgery, it was combined with EuroSCORE in this study to further improve the model’s predictive power. Considering that single-score assessments are inadequate in elderly patient groups with multiple cardiovascular comorbidities, an integration with improved consistency and reliability was created to increase predictive power in different patient populations.

### Data cleaning and pre-processing

The most appropriate parameters for the study plan were determined, and interviews were conducted with the hospital information system teams at Istanbul Medipol Mega University Hospital to ensure that preoperative risk factors were available. New parameters were added or removed according to the experts’ assessments. Therefore, the database of the study was formed from risk factors selected based on reliability, objectivity, and prevalence using data derived from our own research [14]. In this study, each variable’s percentage of missing data was calculated, and features with more than 20% missing data were excluded from the analysis to enhance prediction accuracy [15]. Variables that were deemed unreliable or did not contribute significantly to the model were also eliminated from the analysis. For instance, the Ventricular Septal Defect (VSD) variable, with its low prevalence (only one out of 543 patients having VSD), and its peripheral relevance to the study’s primary focus on non-congenital conditions was excluded from the analysis.

Missing values for categorical variables were imputed using the mode, whereas missing values for numerical variables were filled using the median. These imputations were performed using the mode and median values of the entire dataset to ensure consistency [16]. The dataset variables were classified into categorical and numerical (continuous) types based on the data types. Categorical variables were processed using one-hot encoding, converting non-numeric categories into numerical forms that ML models can interpret. Numerical variables, such as Age, LVEF, and CR, were scaled from 0 to 1 using the Min-Max Scaler. This scaling ensures that variables with different units of measurement become comparable in analytical models, thus enhancing the accuracy and efficiency of model training [10]. the exclusion of features due to missing data may overlook critical information. If these excluded features are deemed essential, then methods for imputing missing data should be considered. Therefore, expert consultation was conducted to assess the importance of these features. Only after reaching a consensus with the experts were the features excluded from the analysis, ensuring that the decision was informed and justified.

### Baseline statistical analysis

Continuous variables were expressed as mean ± standard deviation (SD) for normally distributed data and as median with interquartile range for non-normally distributed data. Appropriate statistical tests, such as Student’s t-test or the Mann–Whitney U test, were used to compare continuous variables. Categorical variables were presented as frequencies (percentages) and compared using Chi-square test or Fisher exact test. The Shapiro-Wilk test was used to evaluate the normality of the variables. Additionally, differences in AUROC values between models were statistically assessed using DeLong’s test. All p-values were two-tailed, with p 0.05 considered statistically significant.

### Model development

**Fig 1:**
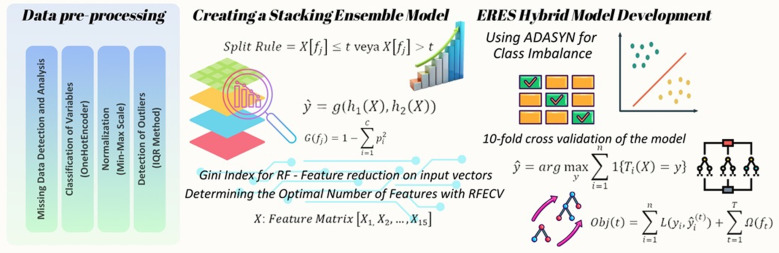
ERES hybrid model development process integrating machine learning models and feature selection.

In this study, a comprehensive modeling process was conducted to predict the postoperative mortality risk following cardiac surgery using various ML algorithms. The primary outcome of interest was postoperative mortality, defined by the “MR” target variable within the preoperative dataset, according to the STS Adult Cardiac Surgery (version 2.81) guidelines [17]. The dataset was randomly split into a training set (70%) and a testing set (30%) using a 7:3 ratio via the train_test_split function (random_state = 42). The models employed during training included Logistic Regression (LR) (max_iter = 3000), Random Forest (RF), Support Vector Machine (SVM) (kernel = ‘linear’, decision_function_shape=’ovr’, probability = True), Multilayer Perceptron (MLP) neural network (max_iter = 3000), Decision Tree (DT), and Extreme Gradient Boosting (XGBoost) (eval_metric = ’mlogloss) [17–23]. As shown in Table 1, 23 variables were used as model inputs. However, as explained in the Characteristics of Patients section, BMI and VSD variables were removed due to specific reasons. The target variable was MR. To enhance the performance of the ML models, the two best-performing models, RF and XGBoost, were combined into a stacking ensemble (SE) model. Because clinical decision-making requires physicians to review a patient’s medical history and current examination results, this process can lead to time loss and slow down real-time decision-making. Therefore, feature selection (FS) is a crucial preprocessing step prior to model prediction. By employing ML algorithms to eliminate irrelevant factors, clinical decision errors can be reduced and accuracy can be improved [24]. Thus, for the best-performing RF model, the most important features were selected using Gini importance, which measures the contribution of each feature to the model’s decision-making process [25]. Following feature selection, RFECV (Recursive Feature Elimination with Cross-Validation) was applied to determine the optimal number of features that would maximize the model’s performance. RFECV uses cross-validation to automatically identify the number of features that provide the best performance [26]. After applying feature selection methods, the 15 most predictive features were identified, and the ERES model was trained using these features. To address class imbalance in the dataset, the ADASYN method was employed, which generates synthetic samples for the minority class to balance the dataset. This method was selected to improve the performance of classification models on imbalanced datasets [10]. The resulting set of 15 selected features was then used to develop the SE model. This hybrid model was developed using ADASYN for data balancing, the SE approach, and 10-fold cross-validation [19,27,28]. To enhance feature reduction, this study introduces and validates a hybrid algorithm that integrates machine learning-based feature selection methods before classification using RF (Fig 1). The resulting hybrid model was named the Ensemble-Based Risk Estimation System (ERES).

**Table 1:** Baseline characteristics of the study participants.

	Description	Study Population (n = 543)	*p*-Value	Missing Value Percentage (%)
**Age, years**	Age (in years)	63.87 ± 11.64	<0.05^a^	0.00
**LVEF**	Left Ventricular Ejection Fraction	55.00 (50.00, 60.00)	<0.05^a^	16.39
**CR**	Creatinine (mg/dL)	1.09 (0.86, 1.57)	<0.05^a^	3.31
**BMI**	Body Mass Index	28.50 (27.89, 29.34)	< 0.2199	41.99
**Sex, n**	Female	182 (33.52%)	0.0168	0.00
**SP**	Surgery Priority; 0- Elective, 1-Emergency	63 (11.60%)	<0.05^a^	5.71
**DM**	Diabetes Mellitus	194 (35.73%)	0.0047	0.00
**CVA**	Cerebrovascular Accident or Stroke	17 (3.13%)	0.0399	0.00
**IHD**	Ischemic Heart Disease	367 (67.59%)	0.7726	0.00
**ASCVD**	Atherosclerotic Cardiovascular Diseases	311 (57.27%)	<0.05^a^	0.00
**LD**	Liver Disease	5 (0.92%)	0.0511	0.00
**ND**	Neurological Disorder	15 (2.76%)	1.00	0.00
**COPD**	History of Chronic Obstructive Pulmonary Disease	36 (6.63%)	0.0032	0.00
**CS**	Cardiogenic Shock	25 (4.60%)	<0.05^a^	0.00
**PEI**	Previous Endotracheal Intubation	14 (2.58%)	<0.05^a^	0.00
**CPR**	Previous Cardiopulmonary Resuscitation	29 (5.34%)	<0.05^a^	0.00
**MI**	The recent Myocardial Infarction	71 (13.08%)	<0.05^a^	0.00
**UAP**	Unstable Angina Pectoris	158 (29.10%)	0.0171	0.00
**CHF**	Congestive Heart Failure	115 (21.18%)	<0.05^a^	0.00
**TAS**	Thoracic Aortic Surgery	15 (2.76%)	<0.05^a^	0.00
**VSD**	Post-Infarct Ventricular Septal Defect	1 (0.18%)	1.00	0.00
**RD**	Renal Dysfunction	74 (13.63%)	<0.05^a^	0.00
**ACE**	Active Endocarditis	8 (1.47%)	<0.05^a^	0.00
**PCS**	Reoperation (Previous Cardiac Surgery)	74 (13.63%)	<0.05a	0.00
**PHT**	Pulmonary Hypertension (mmHg)	140 (25.78%)	<0.05^a^	0.00
**MR**	Mortality 0-No and 1-Yes	188 (34.62%)	<0.05^a^	0.00

^a^ p < 0.05; n (%). **Shapiro-Wilk P-Value** (Age, 0.1305; LVEF, CR and BMI, < 0.05a)

The RF model was configured as an ensemble model comprising 100 decision trees (n_estimators = 100). The model constructs each decision tree using random sampling and feature selection, and the final classification is based on the average predictions of these trees. To ensure the reproducibility of the results, the random seed was set to 42 (random_state = 42). The XGBoost model was configured based on the gradient boosting method, where successive decision trees were constructed to correct the errors of the preceding trees. In the XGBoost model, multiclass logarithmic loss was used as the evaluation metric (eval_metric = ‘mlogloss). The random_state parameter was set to 42 to ensure the reproducibility of the model’s random processes. In this hybrid model, the predictions from both base models are fed into the final classifier, the RF model. To robustly assess the model’s performance across various data subsets, we employed 5-fold cross-validation for training and testing phases [29]. The overall performance metrics were evaluated using a 10-fold cross-validation approach [19].

ML-based classification models estimate the probability that each observation in the test set belongs to a particular class. A standard threshold of 0.5 is typically applied to convert these probabilities into binary outcomes: probabilities of 0.5 or higher are classified as “survived,” while those below 0.5 are classified as “deceased”. However, in scenarios with a high mortality rate, this default threshold can weaken the model’s predictive performance because most patients are classified as “deceased.” Therefore, to create distinct clinical risk groups, the population was dichotomized based on the ML risk score, and the optimal threshold for event prediction was determined using the Youden index [26].

### Assessment of model performance

In this study, the performance of various ML models, including LR, RF, SVM, MLP, DT, XGBoost, EuroSCORE I, and the newly developed ERES models, was comprehensively assessed using multiple evaluation metrics. The assessment began with the calculation of key performance metrics for each model, including accuracy, precision, recall, F1 score, and Receiver Operating Characteristic Area Under the Curve (ROC AUC) [30]. The Brier Score was also computed to evaluate the calibration of each model, while Precision-Recall curves and Average Precision scores were generated to assess the models’ ability to distinguish between classes in an imbalanced dataset [21,31]. To gain further insights, ROC curves were plotted for all models, highlighting the trade-off between sensitivity and specificity. The ROC AUC provides a summary of the overall performance. Calibration curves were also plotted to evaluate the alignment between predicted probabilities and actual outcomes, offering insights into the reliability of the models’ probability predictions [32]. For the ERES model, 10-fold cross-validation was performed to ensure the robustness of its performance across different data subsets. This process involved training and validating the model on 10 different splits of the dataset, with mean and 95% confidence intervals (CI) calculated for each metric, ensuring a rigorous evaluation of the model’s consistency and reliability [32]. Furthermore, the Precision-Recall curve and Area Under the Precision-Recall Curve (AUPRC) were calculated, particularly focusing on the ERES model [19]. This analysis was crucial for understanding model performance in scenarios where the data is imbalanced, as the Precision-Recall curve provides more detailed insights than the receiver operating characteristic curve. Bootstrap resampling was employed to validate the Area Under the ROC curve (AUROC) scores of the EuroSCORE I and ERES models. By generating 1000 resampled datasets, the distribution of AUROC scores was estimated, thereby providing robust statistical comparison between the models. An Analysis of Variance (ANOVA) test was subsequently performed to determine whether there were statistically significant differences in the mean AUROC values across the models, with a p-value 0.05 indicating significant differences [6]. In addition, SHAP (SHapley Additive exPlanations) values were calculated for the XGBoost and final estimator of the stacking model to further enhance the interpretability of the models [33]. The robustness of the stacking model was verified by fitting it to the resampled dataset, ensuring that the model was properly trained. Following the SHAP analysis, decision curve analysis (DCA) was performed to evaluate the clinical utility of the models, particularly focusing on the ERES model. DCA offered a detailed assessment of the net benefit provided by the model at various threshold probabilities, ensuring that the ERES model not only had predictive accuracy but also offered practical utility in clinical decision-making [18,23]. This comprehensive approach to model evaluation, combined with SHAP and DCA analysis, ensured that the ERES model was both accurate and interpretable, providing a reliable tool for assessing mortality risk in clinical settings [21]. The comparison and creation of ML models were performed using PyCharm version 2023.3.5 in Python version 3.12. Additional analyses and figure creation were performed using the following Python packages: SciPy version 1.12.1, pandas version 2.2.1, NumPy version 1.26.4, scikit-learn version 1.5.1, imbalanced-learn version 0.10.1, seaborne version 0.12.2, and plotlib version 3.7.1.

## Results

### Characteristics of patients

The baseline characteristics of the study participants (n = 543) are detailed in Table 1. The mean age of the participants was 63.87 ± 11.64 years, with a significant distribution difference (*p* < 0.05). The median LVEF value was 55.00% (IQR 50.00, 60.00), with 16.39% of values missing. The median CR level was 1.09 mg/dL (IQR 0.86, 1.57), and the median BMI was 28.50 kg/m² (IQR 27.89, 29.34), with 41.99% of BMI values missing. The cohort was predominantly male, with 33.52% females (*p* = 0.0168). Significant differences were observed in various clinical conditions, including DM (35.73%, *p* = 0.0047), COPD (6.63%, *p* = 0.0032), CS (4.60%, *p* < 0.05), and RD (13.63%, *p* < 0.05). Additionally, the study found that 34.62% of the population experienced mortality (*p* < 0.05). The Shapiro-Wilk test indicated a non-normal distribution of LVEF, CR, and BMI (*p* < 0.05), requiring non-parametric statistical methods for these variables. These baseline characteristics highlight the clinical complexity and heterogeneity of the study population.

### Comparison of models’ performance

**Fig 2:**
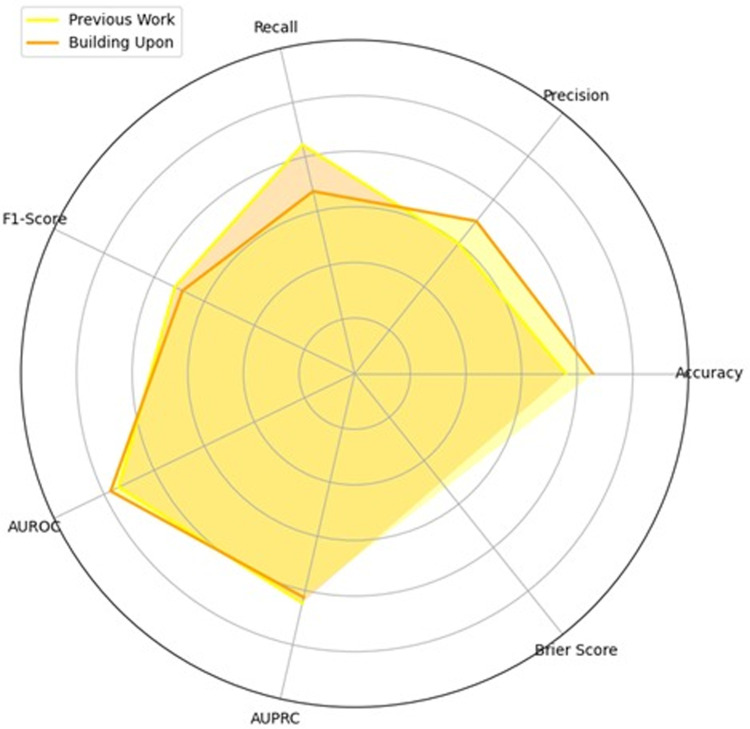
Comparison of performance metrics between previous and current studies.

Building on our previous study, we present a comparative graph (Fig 2) that illustrates the differences between the earlier work and the current research. This visual comparison highlights the improvements achieved in predicting postoperative mortality risk, demonstrating that our models outperform those from prior studies (see S1 Table for detailed metrics of previous models). Fig 2 specifically compares the average performance metrics across the two studies, focusing on ML models used for postoperative mortality prediction. The chart underscores the advancements made in the current study (denoted as “Building Upon”), showing enhanced performance across most metrics relative to the earlier work. Overall, this visual representation emphasizes that the current models have effectively expanded upon the foundation laid by previous research, leading to more accurate and reliable mortality predictions in postoperative contexts.

**Fig 3:**
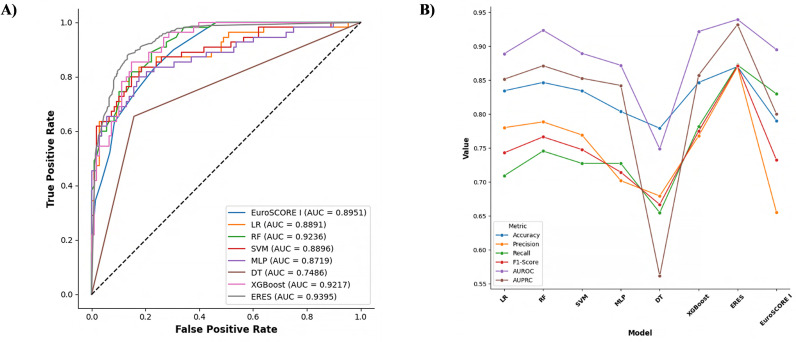
AUC curves (A) and model performance comparisons (B).

Ensemble models, a widely used technique in ML, enhance model performance by combining multiple models [30]. This approach leverages the strengths and mitigates the weaknesses of different models with the goal of creating robust and generalizable prediction systems. Given the performance differences in individual ML models across various datasets, employing multiple models in an ensemble typically leads to improved outcomes. In particular, evaluating the AUROC results using the bootstrap method provides crucial guidance for selecting the models to be included in the ensemble [34]. In this context, the performances of the RF and XGBoost models were statistically validated using an ANOVA test. The RF and XGBoost models exhibited higher AUROC values compared to other ML algorithms, with the differences being statistically significant. According to the ANOVA results, a significantly low p-value (*p* < 0.05) indicates that there are statistically significant differences in the AUROC values between the models. This confirms that the performance differences are not due to random variation; rather, they reflect the true performance disparities between the models. The RF model demonstrated the highest performance with an average AUROC value of 0.9236. This model uses a large number of decision trees, thereby promoting diversity among the trees and reducing the tendency for overfitting. The ability of the RF to generalize data makes it one of the top-performing classification models. The XGBoost model, with an AUROC of 0.9217, demonstrated performance that was close to that of the RF model. By employing gradient boosting, XGBoost has a faster learning capacity, thereby producing effective and rapid results on large datasets. In addition, it offers advanced parameter-tuning techniques, allowing for more efficient model optimization. The high AUROC values of these two models and the statistical significance of the p-value clearly indicate that RF and XGBoost should be included in the stacking ensemble (SE) model. The ROC curve analysis demonstrates the comparative performance of various models in predicting postoperative mortality risk (Fig 3A). The ERES model exhibited the highest discriminative ability with an AUC of 0.9395, outperforming all other models, including the traditional EuroSCORE I model, which achieved an AUC of 0.8951. Among the ML models, RF and XGBoost also showed strong predictive capabilities, with AUC values of 0.9236 and 0.9217, respectively. LR and SVM displayed comparable performance, with AUCs of 0.8891 and 0.8896, while the MLP model yielded a slightly lower AUC of 0.8719. The DT model, which has an AUC of 0.7486, demonstrated the weakest predictive performance. These findings underscore the enhanced accuracy and reliability of the ERES model, particularly when compared with the established EuroSCORE I and other traditional ML models, in predicting postoperative mortality.

The comparative analysis of the models, including ERES, revealed its superior performance across multiple metrics, as illustrated by the ROC curve analysis (Fig 3B). ERES achieved the highest AUROC value of 0.9395, surpassing both the individual models and the traditional EuroSCORE I, which had an AUROC of 0.8951. The detailed performance metrics indicate that ERES model exhibited the highest accuracy (0.8699), precision (0.8698), and recall (0.8722), followed by XGBoost with slightly lower values. Other models like SVM and MLP also performed well, albeit with moderately lower scores. Importantly, the ANOVA test conducted on the AUROC values revealed a statistically significant difference (*p* < 0.05) between the groups, highlighting the enhanced discriminative ability of the ERES model compared with the others (Table 2). The average metric values for both previous and current models are summarized in S2 Table.

**Table 2:** Comparative performance metrics of ML models and risk scores for postoperative mortality prediction.

	LR	RF	SVM	MLP	DT	XGBoost	EuroSCORE I	ERES
**Accuracy**	0,8344	0,8282	0,8344	0,8221	0,8037	0,8466	0,7901	0,8699
**Precision**	0,7800	0,7547	0,7692	0,7241	0,7091	0,7679	0,6555	0,8698
**Recall**	0,7091	0,7273	0,7273	0,7636	0,7091	0,7818	0,8298	0,8722
**F1-Score**	0,7429	0,7407	0,7477	0,7434	0,7091	0,7748	0,7324	0,8710
**AUROC**	0,8891	0,9236	0,8896	0,8719	0,7486	0,9217	0,8951	0,9395
**AUPRC**	0,8514	0,8526	0,8528	0,8574	0,6010	0,8574	0,8001	0,9320
**Brier Score**	0,1163	0,1110	0,1153	0,1473	0,1963	0,1259	0,2099	0,0972

Because of the ANOVA test for the AUROC value, p < 0.05 was found, indicating a statistically significant difference between the groups.

### Feature selection and importance

**Fig 4:**
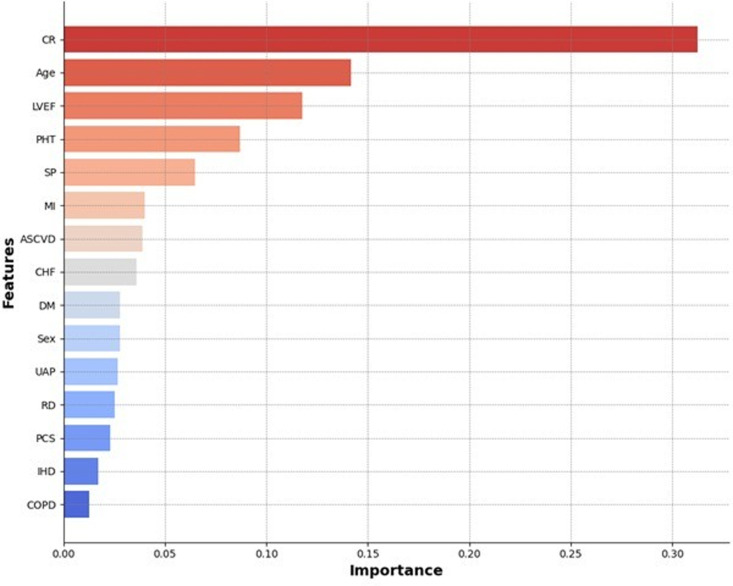
Key predictors of Gini importance.

The feature importance graph generated using the Gini importance metric and RFECV for the RF model provides valuable insights into the relative contribution of each feature in predicting postoperative mortality. The chart highlights that CR is by far the most influential predictor, with a significantly higher importance score than the other features (Fig 4). This finding is consistent with clinical knowledge, as elevated creatinine levels are often associated with poorer outcomes in cardiac surgery patients owing to impaired renal function [26].

Age is the second most important feature, which is consistent with its well-documented role as a critical risk factor for surgical outcomes. LVEF and PHT are also prominent predictors of age, underscoring the importance of cardiac function and pulmonary status in determining patient risk [10]. Other features, such as SP, MI, and ASCVD, also contribute to the model to a lesser extent. These features reflect the complexity of preoperative risk assessment, in which a combination of comorbidities and patient characteristics collectively influence outcomes. The lower-ranking features, such as CHF, DM, and sex, although less critical according to the Gini importance, still play a role in the overall risk prediction, albeit to a lesser degree. The importance of each feature in the model supports the notion that a multifactorial approach is essential for accurately predicting postoperative mortality risk.

### ERES model performance and validation

The ERES model’s performance in predicting postoperative mortality was rigorously evaluated through three critical analyses: calibration, overall model performance with confidence intervals, and precision recall assessment. Each analysis provides important insights into the model’s accuracy and reliability.

**Fig 5:**
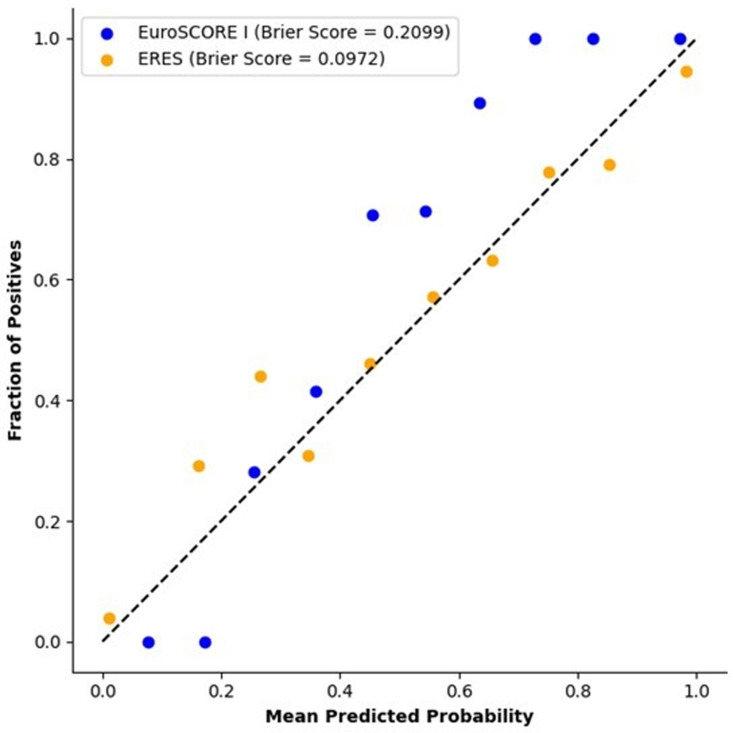
Calibration curves comparing EuroSCORE I and ERES models with Brier scores.

The calibration curve provides a visual comparison of the EuroSCORE I and ERES models in terms of how well their predicted probabilities match the observed outcomes (Fig 5). The ERES model, which has a Brier score of 0.0972, shows a closer alignment with the ideal calibration line than the EuroSCORE I model, which has a Brier score of 0.2099. The ERES model not only predicts mortality risk with higher accuracy but also provides more reliable probability estimates.

**Fig 6:**
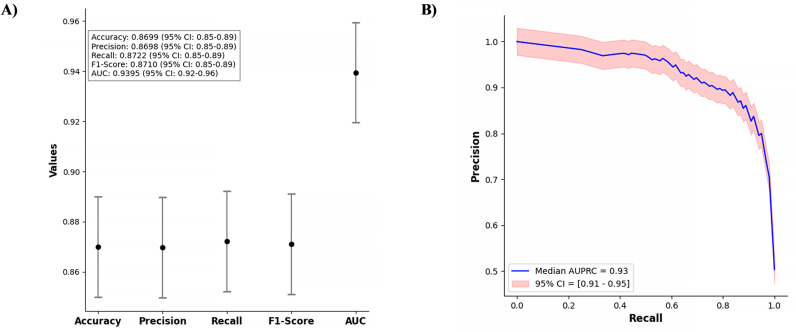
Performance of the ERES model with 95% confidence intervals (A) and precision-recall curve for ERES model (B).

The overall performance of the ERES model is presented, displaying the model’s key metrics—accuracy, precision, recall, F1-score, and area under the curve (AUC)—each presented with 95% confidence intervals. The model achieved an accuracy of 0.8699, precision of 0.8698, recall of 0.8722, an F1-score of 0.8710, and an AUC of 0.9395. These metrics confirmed the model’s robust performance and reliability in predicting postoperative mortality (Fig 6A). The precision-recall curve further validates the effectiveness of the ERES model, with a median AUPRC of 0.93 and a 95% confidence interval ranging from 0.91 to 0.95. This curve highlights the model’s ability to accurately identify high-risk patients, with a strong balance between precision and recall, which is critical in clinical decision-making (Fig 6B).

**Fig 7:**
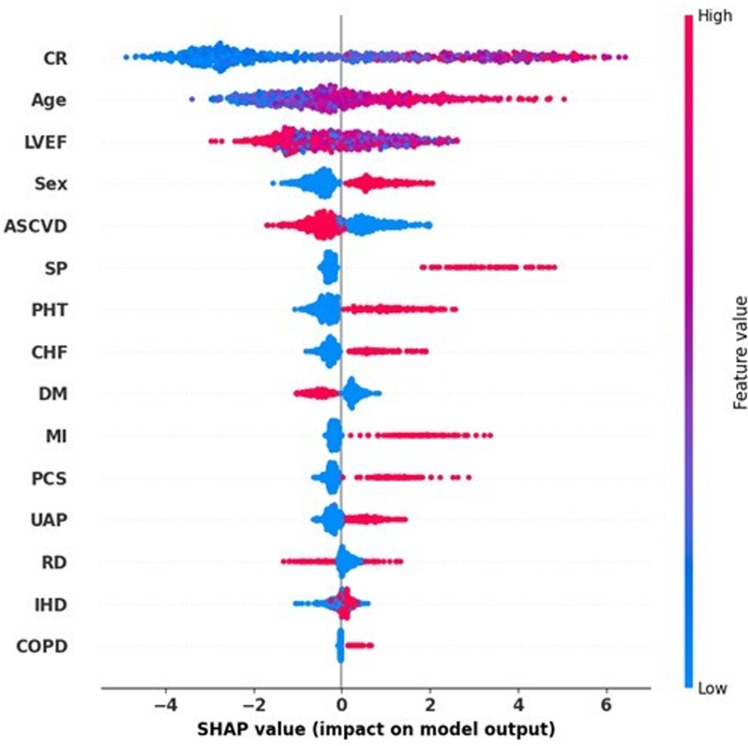
Contributions of input features to mortality prediction in the ERES model.

The SHAP analysis identifies key features such as CR, Age, and ASCVD that exhibit significant positive SHAP values (Fig 7). These findings indicate a strong association between higher levels of these variables and an increased risk of mortality. This analysis highlights the importance of these clinical factors in the model’s predictions, providing valuable insights into the underlying drivers of patient outcomes. Elevated creatinine levels, which indicate kidney dysfunction, are particularly correlated with a higher mortality risk, as shown by the concentration of red points on the positive side of the SHAP axis. Similarly, increased age is associated with higher mortality risk, emphasizing the importance of managing these parameters in clinical practice.

In contrast, features like LVEF display predominantly negative SHAP values for higher measurements, indicating that a higher LVEF, which signifies better heart function, is associated with a greater likelihood of survival. This finding is consistent with clinical understanding, as patients with higher LVEF are less likely to experience severe cardiovascular events that could lead to mortality. Additionally, PHT and ASCVD are significant factors, and higher values tend to increase the probability of mortality. This indicates that both pulmonary hypertension and atherosclerotic cardiovascular disease are critical in determining patient outcomes, underscoring the need for targeted therapeutic strategies in patients with these conditions. Regarding SP, although SP was classified into emergency and elective categories, the SHAP values in the graph indicate that its impact is less pronounced compared to variables like CR, Age, and LVEF. However, a more detailed classification and accurate recording of surgical priority in patient records could lead to better risk stratification. By refining the categories within the Surgery Priority variable, it may become possible to capture the nuances of surgical risk, leading to more precise risk predictions and improved patient management strategies.

### Clinical utility and model interpretation

**Fig 8:**
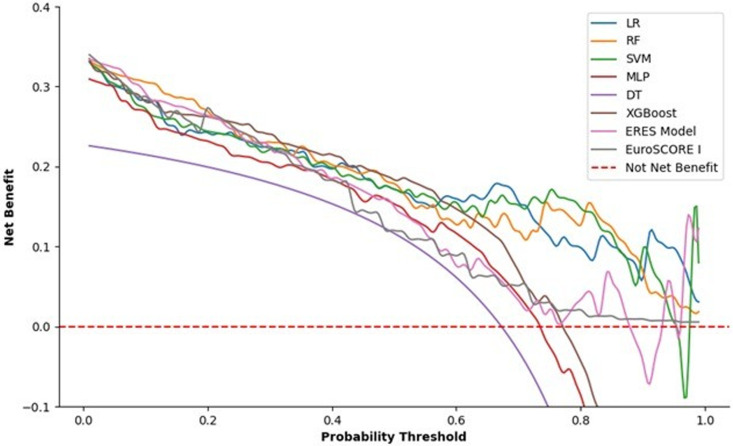
Decision curves illustrating the clinical utility of EuroSCORE I, ERES, and ML models in forecasting postoperative mortality are presented.

In medical decision-making, especially in the context of predicting mortality risk following cardiac surgery, assessing the performance of prediction models is essential for balancing the benefits and risks of medical interventions. Traditional metrics like sensitivity and specificity are useful but often inadequate when evaluating the real-world implications of clinical decision thresholds. DCA offers a more practical evaluation by estimating the net benefit of different models across a range of probability thresholds [32]. Among the models, the ensemble stacking classifier (ERES Model) demonstrated one of the most stable performances across various thresholds, as shown in the DCA analysis in Fig 8. EuroSCORE I, a well-known clinical risk scoring system, also perform competitively, but its net benefit declines sharply at higher thresholds. These results indicate that EuroSCORE I may be less effective when applied to high-risk cases than more flexible ML models. The red dashed line on the DCA plot at zero net benefit represents the scenario where no patients are operated on or all patients are operated on—indicating the baseline for decision-making. Any model above this line provides a tangible advantage over a “treat all” or “treat none” approach.

This DCA emphasizes the superiority of ML models, particularly RF and ensemble methods like the ERES Model, in predicting mortality risk in patients undergoing cardiac surgery. Compared to traditional scoring systems like EuroSCORE I, these models offer enhanced decision-making support by maintaining higher net benefits across thresholds.

### Dual analysis of the ASA classification and EuroSCORE models

**Fig 9:**
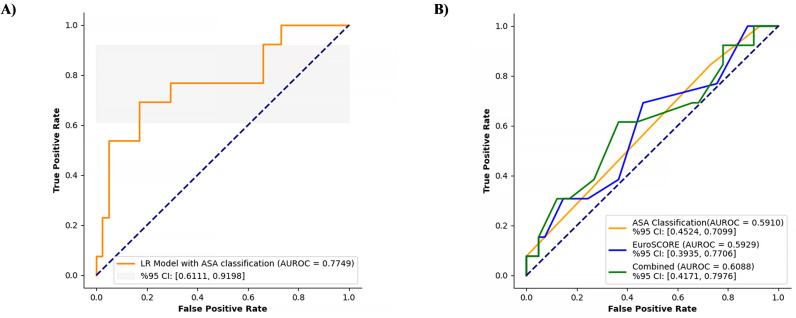
ROC Curve for mortality prediction using ASA classification (A) Mortality prediction performance: ASA, EuroSCORE, and combined models. (B).

The discriminative abilities of the models were evaluated using the AUROC metric. The overall AUROC was calculated as 0.7749, with a 95% CI ranging from 0.6111 to 0.9198 (Fig 9A). This indicates a good level of discrimination, which indicates that the model can effectively distinguish between the positive and negative classes. However, the ASA model yielded a lower AUROC of 0.5910 (95% CI: 0.4524–0.7099), whereas the EuroSCORE model produced a similar AUROC of 0.5929 (95% CI: 0.3935–0.7706) (Fig 9B). Both models demonstrated limited discriminative power because their AUROC values were close to 0.5, indicating that their performance was comparable to that of random chance. The combined model achieved a slightly higher AUROC of 0.6088 (95% CI: 0.4171–0.7976), yet it still exhibited limited discriminative capability. A DeLong test was conducted to compare the AUROC values of the ASA and EuroSCORE models, yielding a DeLong statistic of -0.0198 and a *p*-value of 0.9841. Because the *p-*value exceeds the conventional threshold of significance (*p* > 0.05), we conclude that there is no statistically significant difference between the performances of the two models. Comprehensive statistical analyses of both categorical and continuous variables in the ASA dataset are summarized in S3 and S4 Tables, and the correlation matrix of continuous variables is visualized in S1 Fig.

## Discussion

The results confirm the superiority of ML-based models, specifically the SE approach combining RF and XGBoost, in predicting postoperative mortality risk in patients undergoing cardiac surgery. This finding is consistent with prior research, such as Huang et al., who demonstrated the effectiveness of ML techniques like XGBoost in improving mortality predictions for CABG patients compared with traditional scoring methods [1]. Similarly, Dong et al. highlighted the ability of ensemble methods to outperform individual models when integrating legacy risk scores like EuroSCORE. Their study, while highlighting the benefits of ensemble models, primarily focused on incorporating pre-existing clinical scoring systems, whereas our approach utilizes a feature selection-driven ML framework that optimizes the most predictive clinical variables. By leveraging Gini importance, RFECV, and ADASYN-based balancing, ERES provides a more refined and regionally adapted risk estimation model that enhances both accuracy and clinical utility [30]. In contrast to traditional static models like LR or linear models, our hybrid model incorporates nonlinear interactions. Similar to the approach proposed by Zhang et al., our model addresses imbalanced data using the ADASYN method, which enhances its predictive capabilities [10]. Both studies demonstrated the importance of ML techniques, such as XGBoost, in accurately predicting complications and risks, particularly in imbalanced datasets. Similarly, Lapp et al. utilized ensemble methods, including Random Forest, AdaBoost, and Gradient Boosting, to predict post-cardiac surgery complications, demonstrating AdaBoost’s superiority (AUC = 0.731) over traditional risk scores. However, their approach lacked XGBoost integration and imbalanced data handling, which our study addresses through a stacking ensemble (RF + XGBoost) and ADASYN-based balancing, leading to superior predictive performance (AUC = 0.9395). Furthermore, SHAP and DCA analyses enhance interpretability and clinical applicability, distinguishing our model as a more robust and regionally adapted risk prediction tool [35]. Similarly, Xu et al. employed a bagging-based ensemble approach for CABG mortality prediction, improving accuracy over traditional models. However, unlike our stacking ensemble (RF + XGBoost), their model did not incorporate feature selection or address class imbalance, which we mitigated using Gini importance, RFECV, and ADASYN for enhanced predictive performance and clinical applicability [36].

Feature selection was another crucial step in our methodology. Here, we applied Gini importance and RFECV to select the 15 most relevant features for prediction. Similar to previous studies, such as Allyn et al., which emphasized the importance of feature selection in improving model performance for predicting mortality, our study confirms that eliminating irrelevant features enhances model accuracy [23]. For instance, a study by Khalaji et al. also highlighted the significance of feature selection in predicting mortality after CABG, where total ventilation hours and ejection fraction were identified as the most influential factors in their prediction models [37]. Our findings extend this by demonstrating that even after feature selection, combining multiple ML models further enhances performance. Moreover, while Khalaji et al. applied oversampling techniques like SMOTE to handle data imbalance, our use of ADASYN provided similar advantages, allowing our model to effectively address minority class predictions, thereby improving sensitivity and specificity [37]. Taghizadeh et al. conducted a comparative study on the effectiveness of the adaptive neuro-fuzzy inference system (ANFIS) versus logistic regression in predicting mortality after CABG surgery. The study included 824 patients and utilized input variables including age, sex, body mass index, diabetes mellitus, hypertension, blood cholesterol levels, peripheral vascular disease, addiction, smoking status, and history of chronic heart failure. The fuzzy c-means clustering method was employed to develop the fuzzy inference system model, incorporating Gaussian and linear membership functions for both input and output variables. The results revealed that the ANFIS model achieved a sensitivity of 67%, specificity of 97%, and accuracy of 96%, whereas the logistic regression model reported corresponding values of 48%, 89%, and 89% [38]. Our study employs various ML models, including the ERES, Random Forest (RF), and XGBoost models, with ERES achieving superior performance metrics: accuracy of 86.99%, sensitivity of 87.22%, and receiver operating characteristic curve area of 93.95%. While both studies focused on improving postoperative mortality prediction, the integration of advanced ML techniques, such as ADASYN, for class balancing and feature selection methods in our study allowed for enhanced model performance compared with traditional models like logistic regression [39]. The ERES model demonstrated higher reliability and accuracy in predicting patient outcomes, reinforcing the importance of ML in clinical risk prediction.

The inclusion of DCA in our study highlights the practical clinical utility of ML models compared with traditional scoring systems like EuroSCORE I. Weiss et al. demonstrated how institution-specific ML models based on electronic health records (EHRs) can outperform standard risk scores, such as the STS models, in predicting mortality across various cardiac surgery procedures [16]. Similarly, our ERES model, which integrates the XGBoost and RF models, outperformed EuroSCORE I by providing superior net benefits across multiple decision thresholds. This finding aligns with Weiss et al., who reported that XGBoost consistently outperformed traditional risk scores for mortality prediction, particularly when applied to a multi-modal dataset derived from routine EHR data [16].

In addition, Sinha et al., who conducted the largest multicenter comparative analysis of ML models in predicting mortality after cardiac surgery, also reported that ensemble models like XGBoost and RF, demonstrated superior discrimination compared to EuroSCORE II. Both our study and Sinha et al. confirmed that XGBoost had the best overall performance based on the Brier score and showed the greatest net benefit in DCA [32]. Our study extended these findings by demonstrating that incorporating ADASYN to address class imbalance further enhanced the model’s sensitivity and specificity, thus providing better real-time clinical decision-making support. Sinha et al. also noted that although ensemble models improved calibration compared with traditional LR, there remained some calibration drift, especially at higher risk thresholds [32]. In our study, XGBoost showed a similar trend, where calibration was less accurate in predicting higher-risk cases, reinforcing the need for further refinement, especially in high-risk patient groups. Furthermore, our findings corroborate Weiss et al., who emphasized the importance of including multi-modal features in predictive models to improve mortality risk prediction. Weiss et al. employed XGBoost to identify 15 key features, many of which are consistent with those in our model, such as atrial fibrillation and renal failure, demonstrating the utility of ML in accurately predicting mortality risk across different institutions [16]. Our approach also benefited from a data-driven feature selection process using Gini importance and RFECV, thereby enhancing the clinical utility of our ERES model. This comparative analysis underscores that, while ensemble models like XGBoost and RF are powerful tools for improving prediction accuracy and clinical utility, integrating these models with periodic retraining and incorporating additional clinical and radiological data from electronic health records could further enhance their applicability and generalizability across diverse clinical settings. This comparative analysis highlights the advancements made through the hybrid model in this research and its potential to be further refined and validated across larger datasets and diverse populations.

Because our study examines the validity of a new model within the local healthcare system, we decided not to include EuroSCORE II because our dataset supports EuroSCORE I calculations. This decision was guided by the need to avoid erroneous or misleading results due to data incompatibility. By focusing on EuroSCORE I, we aimed to ensure reliable and accurate analysis within the constraints of our data, highlighting the safe boundaries within which we conducted our research. Comparing models using inconsistent data sources could compromise the validity of our findings. Noyez et al. demonstrated that EuroSCORE II, while an advancement in mortality prediction, has been shown to overestimate mortality in high-risk populations and underestimate risk in low-risk cases, potentially introducing bias in our study [40,41]. Moreover, Mastroiacovo et al. observed that EuroSCORE II’s performance was not reliable for estimating risk in urgent or emergency procedures, further supporting our decision to exclude it [42]. Therefore, our choice to perform EuroSCORE I in this study is consistent with maintaining data reliability. As Barili et al. suggested, although EuroSCORE II provides updates, it does not consistently outperform its predecessor for complex surgeries [41].

The ASA physical status was obtained directly from preoperative anesthesia screening records. In this study, we evaluated the characteristics of the ASA classification as an independent risk stratification metric for postoperative medical complications, and the results demonstrated that the classification exhibited poor discriminatory ability. Specifically, the ASA classification was found to be insufficient for predicting postoperative mortality on its own. These findings are consistent with Cuijpers’ series of 108 patients in which the ASA score showed low predictive performance with an AUC of 0.63 [43]. Similarly, factors such as sample size, patient variability, and the use of single-institution datasets were considered limiting factors in predictive power. In a study by Sankar et al., the ASA classification was reported to have moderate discriminatory ability in predicting 30-day in-hospital mortality (AUC 0.69; CI, 0.62–0.76) and myocardial injury (AUC 0.70; CI, 0.65–0.75) [44]. Similarly, Kivrak et al. noted that the ASA classification showed promise in effectively predicting postoperative mortality. However, the ASA classification has been criticized for its high interobserver variability, which can lead to inconsistent risk prediction results [45]. This variability can negatively impact the accuracy of the risk-benefit assessment process and the quality of communication regarding surgical risks to patients. The study by Kork et al. found that the ASA PS score was somewhat effective in predicting postoperative mortality [46]. However, its subjective nature and variability in assessments between observers were noted.

In future studies, we will examine EuroSCORE II once a broader, more comprehensive dataset becomes available, which would not only allow for more accurate risk assessments and enable cross-model comparisons. Such comparisons help identify strengths and weaknesses across different models and ultimately enhance the international applicability and generalizability of our findings. Additionally, expanding the dataset to include diverse patient populations and surgical procedures could provide a more robust evaluation of EuroSCORE II performance across different clinical settings.

One limitation of our study is the potential bias introduced by the retrospective EuroSCORE I calculation. Additionally, the model was not externally validated, which limits the ability to assess its generalizability to other cardiac surgery centers. Moreover, the relatively small sample size may have affected the robustness of the findings and limited the statistical power of the analysis.

## Conclusion

In conclusion, the present study demonstrated the superiority of ML-based models, particularly the ERES, in predicting postoperative mortality risk among cardiac surgery patients. By incorporating feature selection methods, such as Gini importance and RFECV, and by addressing class imbalance through ADASYN, the ERES model achieved higher accuracy and reliability than traditional risk scores like EuroSCORE I. The findings suggest that adopting ML models in clinical practice can significantly improve real-time decision making and patient outcomes. Future studies should assess the long-term clinical impact of these models on patient care and explore their integration into existing clinical workflows to ensure seamless implementation in diverse healthcare environments. Additionally, investigating the cost-effectiveness and practicality of deploying these models on a larger scale in routine clinical practice is crucial. Furthermore, our study highlights that while the ASA PS score can indicate a patient’s overall physical status and potential surgical risks, it would benefit from being combined with more objective and detailed assessments to achieve more accurate predictions of postoperative outcomes. In this context, the ASA classification could contribute more effectively to clinical decision-making processes if integrated with more comprehensive surgical risk assessment models. Such integration enhances the robustness of clinical evaluations and improves the precision of risk stratification, especially in complex surgical cases.

## Supporting information

S1 Table Comparative Analysis of Machine Learning Model Performances for Mortality Prediction (Results from Previous Work). (DOCX)

S2 Table Model Performance Metrics: Comparison of Previous Research and Current Study (Mean Calculated Across Rows for Each Metric). (DOCX)

S3 Table Chi-Square and Fisher’s Exact Test Results for Categorical Variables in the ASA Score Dataset. (DOCX)

S4 Table Descriptive and Statistical Analysis of Continuous Variables in the ASA Score Dataset: Mann-Whitney U and Shapiro-Wilk Test Results. (DOCX)

S1 Fig Heatmap of Correlations Among Continuous Variables in the ASA Score Dataset. (DOCX)
