# Supplementary material for: Machine learning-based hybrid risk estimation system (ERES) in cardiac surgery: Supplementary insights from the ASA score analysis
Source: PLOS Digit Health. 2025 Jun 23;4(6):e0000889. doi: 10.1371/journal.pdig.0000889 (PMC12184902; doi:10.1371/journal.pdig.0000889)
Supplement: S1 Table — (DOCX) [file pdig.0000889.s001.docx]

**S1 Table. Comparative Analysis of Machine Learning Model Performances for Mortality Prediction** (Results from Previous Work)

|  | **LR** | **RF** | **SVM** | **MLP** | **DT** | **XGBoost** |
| --- | --- | --- | --- | --- | --- | --- |
| **Accuracy** | 0,8129 | 0,8129 | 0,7626 | 0,7914 | 0,7338 | 0,7626 |
| **Precision** | 0,7500 | 0,7424 | 0,6812 | 0,7042 | 0,6522 | 0,6667 |
| **Recall** | 0,8276 | 0,8448 | 0,8103 | 0,8621 | 0,7759 | 0,8621 |
| **F1-Score** | 0,7869 | 0,7903 | 0,7402 | 0,7752 | 0,7087 | 0,7519 |
| **AUROC** | 0,9132 | 0,9013 | 0,8891 | 0,9061 | 0,7372 | 0,8776 |
| **AUPRC** | 0,8873 | 0,8742 | 0,8576 | 0,8886 | 0,5956 | 0,8332 |
| **Brier Score** | 0,1203 | 0,1283 | 0,1357 | 0,1474 | 0,267 | 0,1636 |
