# Supplementary material for: Machine learning-based hybrid risk estimation system (ERES) in cardiac surgery: Supplementary insights from the ASA score analysis
Source: PLOS Digit Health. 2025 Jun 23;4(6):e0000889. doi: 10.1371/journal.pdig.0000889 (PMC12184902; doi:10.1371/journal.pdig.0000889)
Supplement: S2 Table — (DOCX) [file pdig.0000889.s002.docx]

**S2 Table. Model Performance Metrics: Comparison of Previous Research and Current Study** (Mean Calculated Across Rows for Each Metric)

| ***Research*** | **Accuracy** | **Precision** | **Recall** | **F1-Score** | **AUROC** | **AUPRC** | **Brier Score** |
| --- | --- | --- | --- | --- | --- | --- | --- |
| **Previous work** | 0,7794 | 0,6995 | 0,8218 | 0,7587 | 0,8708 | 0,8228 | 0,1604 |
| **Building upon** | 0,8282 | 0,7508 | 0,7364 | 0,7431 | 0,8841 | 0,8121 | 0,1353 |

Avg_previous work = np.mean(first_row_data, axis=1) Avg_building upon = np.mean(second_row_data, axis=1)

Here, the axis=1 parameter ensures that the mean is calculated across the rows (i.e., for each metric). Thus, the average of all models was computed for each metric.
