# Supplementary material for: Machine learning-based hybrid risk estimation system (ERES) in cardiac surgery: Supplementary insights from the ASA score analysis
Source: PLOS Digit Health. 2025 Jun 23;4(6):e0000889. doi: 10.1371/journal.pdig.0000889 (PMC12184902; doi:10.1371/journal.pdig.0000889)
Supplement: S3 Table — (DOCX) [file pdig.0000889.s003.docx]

**S3 Table. Chi-Square and Fisher's Exact Test Results for Categorical Variables in the ASA Score Dataset**

| **Variables** | **Frequency** | **Proportion (%)** | ***p-*Value** |
| --- | --- | --- | --- |
| **Male** | 104 | 58,43 | 0,6268 |
| **SP** | 9 | 5,06 | 0,0006 |
| **DM** | 75 | 42,13 | 0,1950 |
| **CVA** | 6 | 3,37 | 0,0892 |
| **IHD** | 108 | 60,67 | 0,0331 |
| **ASCVD** | 107 | 60,11 | 0,1416 |
| **LD** | 2 | 1,12 | 0,5468 |
| **ND** | 7 | 3,93 | 0,4297 |
| **COPD** | 10 | 5,62 | 0,0146 |
| **CS** | 1 | 0,56 | 0,3258 |
| **CPR** | 1 | 0,56 | 0,3258 |
| **MI** | 9 | 5,06 | 0,0059 |
| **UAP** | 51 | 28,65 | 0,0078 |
| **CHF** | 44 | 24,72 | 0,0057 |
| **TAS** | 7 | 3,93 | 0,0380 |
| **VSD** | 0 | 0,0000 | 1,0000 |
| **RD** | 30 | 16,85 | 0,2009 |
| **ACE** | 1 | 0,56 | 0,3258 |
| **PCS** | 22 | 12,36 | 0,0071 |
| **PHT** | 40 | 22,47 | 0,7059 |
| **MR** | 58 | 32,58 | < 0.05^a^ |
| **ASA** | Class 1: 17 | 9.55 | 0.0151 |
|  | Class 2: 29 | 16.29 |  |
|  | Class 3: 126 | 70.79 |  |
|  | Class 4: 6 | 3.37 |  |

a *p < 0.05*
