# Supplementary material for: Machine learning-based hybrid risk estimation system (ERES) in cardiac surgery: Supplementary insights from the ASA score analysis
Source: PLOS Digit Health. 2025 Jun 23;4(6):e0000889. doi: 10.1371/journal.pdig.0000889 (PMC12184902; doi:10.1371/journal.pdig.0000889)
Supplement: S4 Table — (DOCX) [file pdig.0000889.s004.docx]

**S4 Table. Descriptive and Statistical Analysis of Continuous Variables in the ASA Score Dataset:** Mann-Whitney U and Shapiro-Wilk Test Results

|  | **Age** | **BMI** | **LVEF** | **CR** | **EUS** |
| --- | --- | --- | --- | --- | --- |
| **Count** | 178 | 178 | 178 | 178 | 178 |
| **Mean** | 63,8034 | 28,5476 | 51,236 | 1,7546 | 5,5112 |
| **Std** | 11,2114 | 4,3248 | 13,2294 | 3,4256 | 3,2456 |
| **Min** | 29 | 15,92 | 15 | 0,2600 | 0 |
| **25%** | 57 | 27,4 | 41,25 | 0,9525 | 4 |
| **50%** | 65 | 28,4 | 55 | 1,27 | 5 |
| **75%** | 71 | 29,2975 | 60 | 1,8375 | 7 |
| **max** | 92 | 50,67 | 65 | 45,8 | 16 |
| **Mann–Whitney U Test (p-value)** | 0.0045 | 0.8497 | 0.0075 | < 0.05^a^ | 0.0015 |
| **Shapiro-Wilk Test (p-value)** | 0.0064 | < 0.05^a^ | < 0.05^a^ | < 0.05^a^ | < 0.05^a^ |

a *p < 0.05*

The mean age was 63.803 years, the average LVEF was 51.236 years, and the mean EUS was 5.511. This indicates that the patients analyzed were generally middle-aged and had varying cardiovascular risk levels. A significant relationship was found between ASA and MR variables (χ2 = 10.4529, p-value = 0.0151).

The p-values for all numerical variables (Age, BMI, LVEF, CR, and EUS) were below 0.05. This indicates that these variables do not follow a normal distribution, necessitating the use of non-parametric tests. To compare differences between two groups for non-normally distributed variables, the Mann–Whitney U test was used. Significant differences were observed between the mortality and non-mortality groups in Age's variables, LVEF, CR, and EUS. However, no significant difference was found for BMI (p-value = 0.8497), indicating that BMI does not have a pronounced impact on mortality. The Chi-Square test was used to examine the relationship between ASA score and MR. The Chi-Square value was 10.4529, with a p-value of 0.0151, indicating a significant association between the ASA score and mortality.
