# Supplementary material for: Machine learning-based hybrid risk estimation system (ERES) in cardiac surgery: Supplementary insights from the ASA score analysis
Source: PLOS Digit Health. 2025 Jun 23;4(6):e0000889. doi: 10.1371/journal.pdig.0000889 (PMC12184902; doi:10.1371/journal.pdig.0000889)
Supplement: S1 Fig — (DOCX) [file pdig.0000889.s005.docx]

A correlation matrix was computed for the continuous variables (Age, BMI, LVEF, CR, and EUS) and visualized using a heatmap. A positive correlation was observed between Age and EUS (r = 0.31), indicating that as age increases, the EuroSCORE also increases. Additionally, a negative correlation was found between LVEF and EUS (r = -0.36), suggesting that as cardiac function deteriorates (reflected by a decrease in LVEF), the associated risk (EUS) increases.


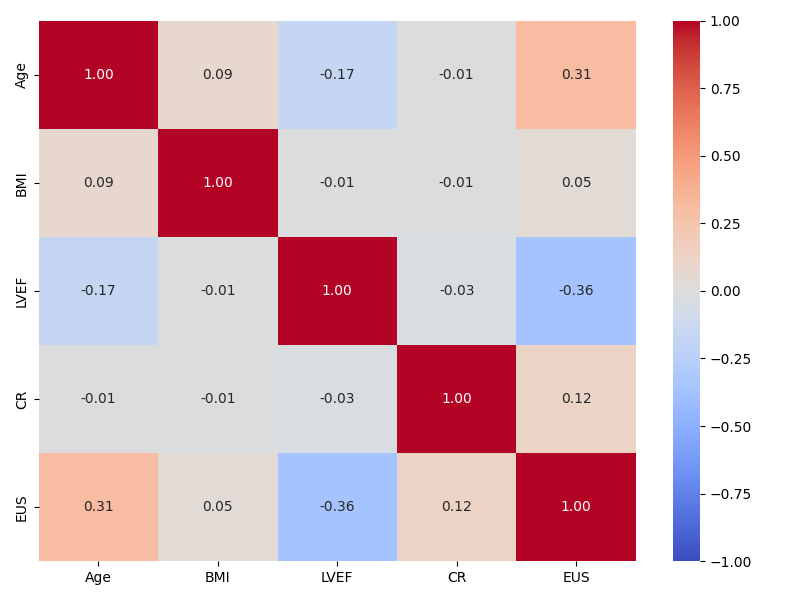


**S1 Fig. Heatmap of Correlations Among Continuous Variables in the ASA Score Dataset**
